# The Significance of the Right Dorsolateral Prefrontal Cortex for Pitch Memory in Non-musicians Depends on Baseline Pitch Memory Abilities

**DOI:** 10.3389/fnins.2017.00677

**Published:** 2017-12-06

**Authors:** Nora K. Schaal, Marina Kretschmer, Ariane Keitel, Vanessa Krause, Jasmin Pfeifer, Bettina Pollok

**Affiliations:** ^1^Department of Experimental Psychology, Heinrich-Heine-University, Düsseldorf, Germany; ^2^Medical Faculty, Institute of Clinical Neuroscience and Medical Psychology, Heinrich-Heine-University, Düsseldorf, Germany; ^3^Amsterdam Center for Language and Communication, University of Amsterdam, Amsterdam, Netherlands; ^4^Institute for Language and Information, Heinrich-Heine-University, Düsseldorf, Germany

**Keywords:** pitch memory, cathodal tDCS, right DLPFC, non-musicians, baseline performance

## Abstract

Pitch memory is a resource which is shared by music and language. Neuroimaging studies have shown that the right dorsolateral prefrontal cortex (DLPFC) is activated during pitch memory processes. The present study investigated the causal significance of this brain area for pitch memory in non-musicians by applying cathodal and sham transcranial direct current stimulation (tDCS) over the right DLPFC and examining the impact on offline pitch and visual memory span performances. On the overall sample (*N* = 22) no significant modulation effect of cathodal stimulation on the pitch span task was found. However, when dividing the sample by means of a median split of pre-test pitch memory abilities into a high and low performing group, a selective effect of significantly impaired pitch memory after cathodal tDCS in good performers was revealed. The visual control task was not affected by the stimulation in either group. The results support previous neuroimaging studies that the right DLPFC is involved in pitch memory processes in non-musicians and highlights the importance of baseline pitch memory abilities for the modulatory effect of tDCS.

## Introduction

Pitch perception and memory are important resources in order to process, understand and produce music and speech. Functional neuroimaging studies have identified a complex neural system of frontal, temporal and parietal areas for pitch memory (Zatorre et al., 1994; Gaab et al., 2003; Koelsch et al., 2009; Jerde et al., 2011). For example, the study by Gaab et al. (2003) investigated the neural network of pitch memory in non-musicians using functional magnetic resonance imaging (fMRI) and highlighted activation of the left inferior frontal gyrus, bilateral superior temporal gyri, posterior dorsolateral frontal regions, superior parietal regions, cerebellar lobes V and VI, and the left supramarginal gyrus. A positron emission tomography (PET) study by Zatorre et al. (1994) showed increased activation in the right frontal and temporal lobes as well as in parietal areas and the insula while participants completed a pitch memory task.

While functional brain neuroimaging studies only provide correlational evidence, non-invasive brain stimulation methods, such as transcranial direct current stimulation (tDCS), are useful tools to investigate causal involvements of particular brain areas for cognitive functions (Nitsche and Paulus, 2001; Antal et al., 2004). The excitability of certain brain areas can be modulated using tDCS and the effects on the behavioral outcome give information about the significance of the stimulated area for the task of interest (Herrmann et al., 2013). Two stimulation modes are distinguished for tDCS, anodal and cathodal stimulation. Typically anodal tDCS facilitates cortical excitability in the targeted area and leads to improved performance, whereas cathodal tDCS was mostly shown to suppress cortical excitability which primes diminished performances (Nitsche and Paulus, 2000; Cohen Kadosh et al., 2010; Ladeira et al., 2011). However, some contrary modulation effects depending on certain factors of the stimulation input (e.g., intensity or duration) have been revealed more recently (Batsikadze et al., 2013; Heimrath et al., 2014; Schaal et al., 2015a).

Looking at tDCS studies investigating the neural basis of pitch memory in non-musicians, the left supramarginal gyrus (SMG) is one area that has received some attention with studies showing that pitch memory was facilitated after anodal stimulation (Schaal et al., 2013, 2017). Cathodal stimulation, on the other hand, led to a deterioration of pitch memory performance in healthy non-musicians (Vines et al., 2006; Schaal et al., 2015b). Taken together these studies provide strong evidence for the significance of the left SMG for pitch memory. They further highlight the potential to explore whether brain areas of interest are involved in the pitch memory process by means of tDCS. To the best of our knowledge, the significance of other brain areas of the underlying neural network of pitch memory in healthy non-musicians using non-invasive brain stimulation methods have not been investigated yet.

Even though the ability to memorize pitches to a certain extent is often taken for granted, about 1.5% of the population have a pitch perception and memory disorder, known as congenital amusia or tone-deafness (Peretz and Vuvan, 2017). Congenital amusia is characterized by a deficient detection of pitch changes (Foxton et al., 2004; Hyde and Peretz, 2004), an impaired discrimination of different pitch directions (Foxton et al., 2004; Liu et al., 2010), and an affected short-term recognition of tone sequences (Williamson and Stewart, 2010; Schaal et al., 2015c). These musical deficits cannot be linked to other cognitive impairments, insufficient exposure to music or a hearing deficiency (Ayotte et al., 2002; Peretz et al., 2002). However, several brain neuroimaging studies identified structural and functional neural differences between amusic individuals and matched controls predominantly in frontal and temporal areas (Hyde et al., 2006, 2007, 2011; Albouy et al., 2013). More specifically and of particular interest for the present study, several studies have shown structural and functional abnormalities in the amusics' brain in the right frontal lobe (Hyde et al., 2006, 2007, 2011). Albouy et al. (2013) conducted a comprehensive study on the neural correlates of impaired pitch memory in congenital amusics using magnetoencephalography and voxel-based morphometry. Anomalies of grey and white matter concentrations in the right inferior frontal gyrus in amusics were shown which supported findings of previous studies (Hyde et al., 2006, 2007). Furthermore, the study revealed that amusics displayed decreased low gamma oscillations (30–40 Hz range) in the right dorsolateral prefrontal cortex (DLPFC) during the retention of pitch information compared to healthy controls.

Based on these findings, a preceding study from our group could show that transcranial alternating current stimulation (tACS) with a gamma frequency of 35 Hz over the right DLPFC in amusics led to increased pitch memory performances. Whereas, the pitch memory performance was significantly impaired without stimulation in amusics compared to healthy controls, the performance was comparable to healthy controls after stimulation (Schaal et al., 2015c). The study therefore proposes a causal link between the functioning of the right DLPFC and pitch memory in congenital amusia. Additionally, it should be noted that studies have shown that amusics display decreased connectivity between the right frontal and temporal lobe (Loui et al., 2009; Hyde et al., 2011). Thus, it is reasonable to assume that the applied stimulation led to an increase of the functioning of the right arcuate fasciculus which combines frontal and temporal areas.

Taken together, studies have highlighted the activation of the right DLPFC during pitch memory in healthy non-musicians (Zatorre et al., 1994; Gaab et al., 2006) and have suggested abnormalities of the right DLPFC as one possible cause for impaired pitch memory abilities in congenital amusia. But a causal significance of the right DLPFC for pitch memory in healthy non-musicians has not been investigated yet. Therefore, the aim of the present study was to investigate the involvement of the right DLPFC for pitch memory in non-musicians. Participants completed a pitch memory task, as well as a visual memory task serving as control condition after receiving either sham or cathodal tDCS over the right DLPFC in two separate sessions. We hypothesized that suppressing the activation of the right DLPFC with cathodal tDCS would lead to a deterioration of pitch memory performance.

## Materials and methods

### Participants

Twenty-seven non-musicians (14 female) with a mean age of 25.3 ± 4.5 years took part in the study. Three participants were excluded as they indicated that they did not understand the span tasks and two data sets were excluded because of a technical error. The final sample consisted of 22 participants (11 female) with a mean age of 25.2 ± 4.2 years. The sample displayed a mean Musical Training Score of the Goldsmiths Musical Sophistication Index (Gold-MSI) of 11.7 ± 4.2 points, confirming that non-musicians took part (Mullensiefen et al., 2014; Schaal et al., 2014). The mean score of the Edinburgh Handedness Inventory (EHI; Oldfield, 1971) was 93.4 (range: 80–100) endorsing that only right-handed participants were included.

The study was approved by the ethics committee of the Medical Faculty of the Heinrich-Heine-University in Düsseldorf (study number: 4044) and was carried out in accordance with the Declaration of Helsinki. All participants signed an informed written consent prior to participation.

### Questionnaires

The Edinburgh Handedness Inventory (EHI; Oldfield, 1971) was included to confirm the right-handedness of the present sample. Each of the 10 items refers to an activity (e.g., writing, throwing, and drawing) and requests participants to indicate their hand preference for performing each activity. The individual responses result in a coefficient for right-handedness varying from 0 to 100%. Participants with a coefficient of 70% and above are classified as right-handed.

In order to ensure that we only included non-musicians participants filled in the German version of the sub-dimension *Musical Training* of the Goldsmiths Musical Sophistication Index (Gold-MSI; Schaal et al., 2014). The Musical Training dimension comprises seven items each to be rated on a 7-point scale from 1 = completely disagree to 7 = completely agree, resulting in a possible range of 7 to 49 points.

A further questionnaire asked participants to indicate what kind of stimulation (anodal vs. cathodal vs. sham) they thought to have received. If participants did not know the meaning of the answering possibilities they were encouraged to guess. This questionnaire intended to determine participants' blindness toward the stimulation mode. Correct estimates were given in 41% of the cases suggesting successful blinding.

### Tasks

Participants completed the same four tasks in a pre-test as well as in the two stimulation sessions. Two of the tasks challenged participants' memory capacities in the auditory or visual modality, respectively. The pitch memory span task was adapted from Williamson and Stewart (2010): The stimulus pool consisted of 10 sine tones each 500 ms in length including 20 ms fading in and fading out. The tones were created in Praat (Boersma and Weenink, 2014) with fundamental frequencies ranging from 262 Hz (C4) to 741 Hz (F#5) in equally tempered, whole tone steps. A constrained random sampling procedure without replacement generated tone sequence pairs from these 10 tones. The two successive sequences of a sequence pair were of equal length and adjacent tones differed by at least four semitones. The inter-tone interval was 383 ms, the inter-sequence interval 2 s after the response was given. In one half of the trials the sequences of each pair were identical. In the other half two tones of the second sequence were presented in the reversed position compared to the first sequence (list probe method). Participants determined whether the two sequences were identical or different and entered their decision via mouse click (two-alternative forced choice paradigm). The sequence length was determined by an adaptive two-up-one-down staircase procedure starting with two-tone sequences. If a participant gave two correct answers to consecutive sequence pairs of length n, the pair presented in the next trial had a length of n + 1. If a participant gave an incorrect answer to one sequence pair, the sequence length for the next trial was decreased by one tone (i.e., reversal). After participants entered their answers they heard a burst of pink noise for 2 s in order to minimize auditory carry-over effects to the next trial. The pitch memory span task ended after four reversals. As a control task we included a visual memory span task (Schaal et al., 2015c) which required participants' memory capacities in the visual modality selectively. This task was developed to match the pitch span task as good as possible. Instead of sine tones black Devanagari letters were presented on a white screen sequentially. These symbols were ranked in similarity from 1 to 10 in order to match the interval relation of the tones from the auditory task (see Schaal et al., 2015c for further information). This ranking was considered during sequence generation to such degree that adjacent letters had at least two similarity steps between them. Analogous to the pink noise burst a checkerboard pattern was presented for 2 s after the participants entered their decisions in order to diminish any visual traces. The initial sequence length was four items in the visual span task. All other aspects of the tasks were identical to the pitch span task.

In addition to memory performances two additional tasks measured participants' sensitivity to discriminate pitches in order to ensure that the intervals used in the pitch memory span task would be supra-threshold for each individual. With regards to psychophysical measures of pitch perception developed by Williamson and Stewart (2010) we evaluated participants' thresholds separately for the detection of a pitch change (pitch detection task) and the discrimination of different directions of pitch glides (pitch direction task). On every trial of both tasks, three pure tones were presented, each with a length of 600 ms, from which one tone—the target—differed from the others. With equal probability the targets were either upward or downward glides centred around 500 Hz. In one half of the trials the target tone was in the first, in the other half in the last position. In the pitch detection task non-targets were steady-state tones of 500 Hz whereas in the pitch direction task the difference between target and non-targets was that the glides went in opposite directions. The task was to identify the target and to enter the decision (first vs. last) via mouse click (two-alternative forced choice paradigm). For both tasks the target of the first trial was set to range six semitones from the non-target tones. Analogous to the memory tasks the difficulty of the perception tasks was individually adapted with a two-up-one-down staircase procedure. In order to increase sensitivity near the individual threshold pitch graduations of the targets became finer as trials proceeded. For each of the first five steps the difference in pitch between target and non-targets was adapted by one semitone. A change of 0.2 semitones was used for steps six to nine and for the last steps the adaption comprised 0.05 semitones. Each task ended after the pitch of the target was adapted 15 times.

### TDCS parameters

The right DLPFC was located using the international 10–20 system for electroencephalogram electrode placement. This method of localization has been reported to be reliable and successful (Herwig et al., 2003; Antal et al., 2004; Schaal et al., 2015b). F4 has been established as a common location for targeting the right DLPFC (e.g., Rossi et al., 2001; Smirni et al., 2015). The active electrode (5 × 5 cm = 25 cm^2^) was placed over F4, the reference electrode (5 × 7 cm = 35 cm^2^) over the left supraorbital area. Both electrodes were covered in saline-soaked sponges and adjusted on the scalp with self-adhesive bandages. Prior to electrode placement the areas were cleaned with alcohol and slightly roughened. A constant-current stimulator (DC-Stimulator, NeuroConn, Germany) delivered a current intensity of 2 mA with a 15-s fade-in and fade-out time. Current density under the active electrode was 0.08 mA/cm^2^. Stimulation was delivered at rest for 15 min in the cathodal stimulation session and for 30 s in the sham stimulation session leading to the sensation of being stimulated in both cases but to relevant influences on cortical excitability in the cathodal stimulation session only. Participants were blinded to stimulation mode.

### Procedure

Participants took part in three sessions (pre-test and two stimulation sessions) separated from each other by one week. The order in which participants completed the four tasks remained constant from session to session, but was counterbalanced across subjects. In total there were six different task orders as the two perception tasks were summarized to one task block consistently starting with the pitch detection task. Each task block took approximately 10 min. Participants listened to the tones of the three auditory tasks via headphones (AKG Pro Audio, K77). All tasks were presented with the computer program Praat (Boersma and Weenink, 2014). For all participants the pre-test served as a practice phase without any stimulation to adapt the volume of the presented tones to an individually comfortable level and to ensure that the tasks were understood. Moreover, the pre-test served as an estimation of the individual performance levels in the respective tasks. Participants received information about the different tasks before each task. After completing the behavioral tasks subjects filled in the EHI as well as the Gold-MSI Musical Training dimension at the end of the pre-test session. The stimulation sessions started with adjusting the electrodes on the scalp and stimulation was then applied for 15 min in which participants remained inactive. The mode of stimulation (sham vs. cathodal tDCS) in the first and second stimulation session was counterbalanced across participants. Immediately after the stimulation participants completed the three task blocks. After every stimulation session they were asked to give an estimate to which mode of stimulation they received.

## Results

### Memory span tasks

For the overall sample, a repeated measures ANOVA with the within-subject factors *stimulation* (sham vs. cathodal tDCS) and *task* (pitch vs. visual memory span task) revealed a significant effect of *task* [*F*_(21)_ = 133.83, *p* < 0.001], a non-significant effect of *stimulation* [*F*_(21)_ = 0.41, *p* = 0.531] as well as a non-significant task^*^stimulation interaction [*F*_(21)_ = 0.60, *p* = 0.449]. Participants displayed significantly longer visual spans (*M* = 7.27 ± 1.54) than pitch spans (*M* = 4.55 ± 1.27). No effect of *stimulation* on either task was revealed. Overall performances on the pitch memory span task were comparable after sham tDCS (*M* = 4.70 ± 1.36) and cathodal stimulation (*M* = 4.39 ± 1.20). The same holds for the visual control task (*M* = 7.25 ± 1.63 after sham and *M* = 7.28 ± 1.47 after cathodal stimulation).

As a next step we divided the sample into two groups by performing a median split on the basis of the pre-test pitch span memory performance receiving a group of participants performing higher than the median of 4 tones (*N* = 11, *M* = 5.89 ± 1.49) and a group displaying lower spans (*N* = 11, *M* = 2.91 ± 0.70). Table 1 gives an overview on basic characteristics of the two groups. A mixed-design ANOVA with the factors *task* (pitch vs. visual span), *stimulation* (sham vs. cathodal) and *group* (below vs. above median pitch span) was calculated. The results revealed a significant main effect of *task* [*F*_(1, 20)_ = 133.66, *p* < 0.001] and a significant task^*^stimulation^*^group interaction [*F*_(1, 20)_ = 8.56, *p* = 0.008]. All other comparisons were non-significant (*p*-values > 0.128).

**Table 1:** Basic characteristics for the overall sample and the two groups based on a median split of the pitch span performance in the pre-test (below and above median).

	**Overall**	**Below median**	**Above median**
Age	25.2 ± 4.2	26.5 ± 4.5	23.9 ± 3.7
Gender (f/m)	11/11	7/4	4/7
Gold-MSI	11.7 ± 4.2	10.3 ± 3.5	13.1 ± 4.4
Pitch Span pre-test	4.40 ± 1.90	2.91 ± 0.70	5.89 ± 1.49
Visual Span pre-test	6.97 ± 1.66	6.87 ± 1.64	7.06 ± 1.76

**Figure 1:**
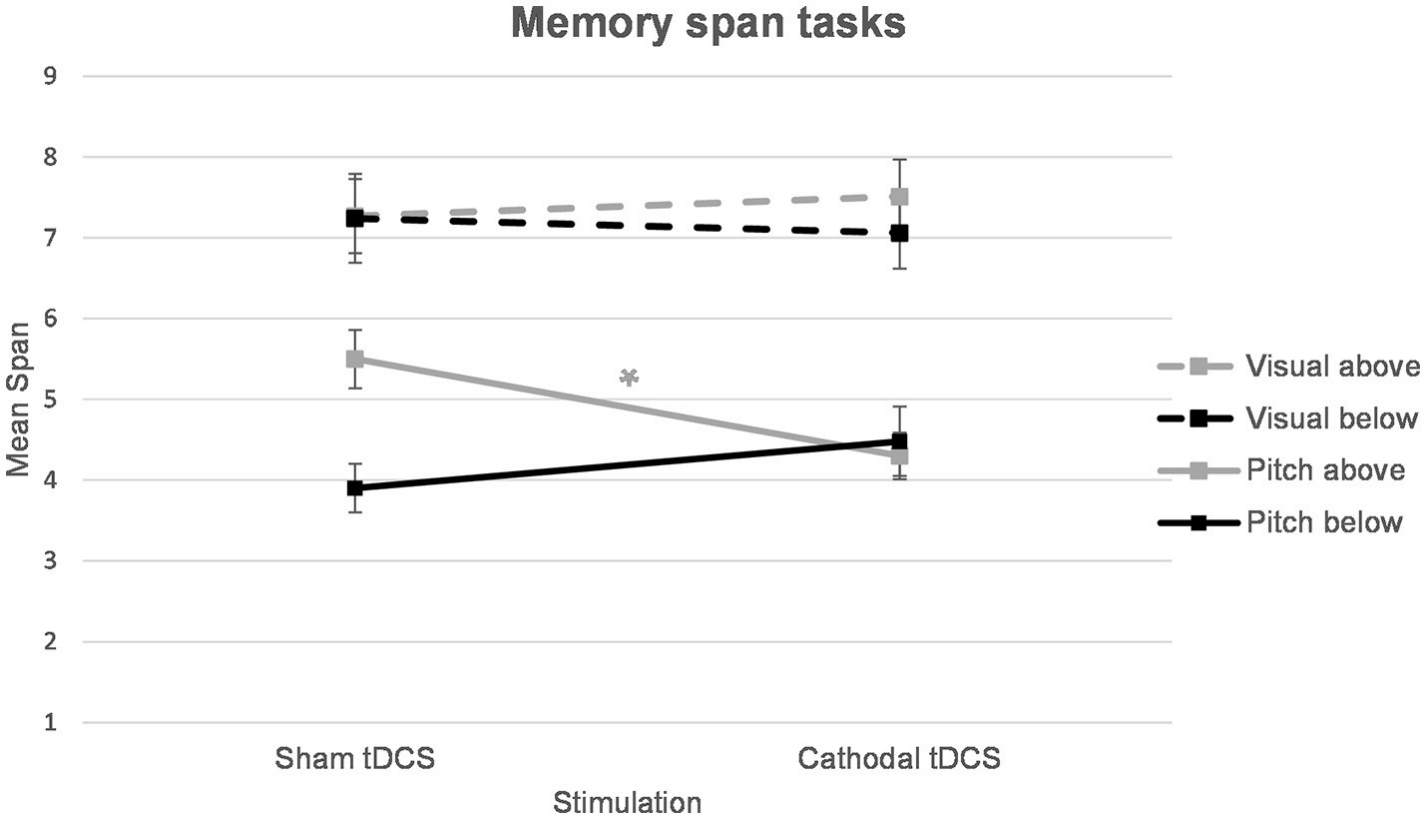
Pitch and visual memory span performances for the two groups (below and above median pitch span performance). A significant deterioration in pitch memory was revealed for good pitch memory performers (above median split group) after cathodal tDCS. ^*^p = 0.014.

In order to disentangle the significant task^*^stimulation^*^group interaction, separate ANOVAs for each task were calculated. For the pitch memory span task, a mixed-design ANOVA with *stimulation* (sham vs. cathodal) as the within-subject factor and *group* (above vs. below median pitch span) as the between-subject factor showed a significant stimulation^*^group interaction [*F*_(1, 20)_ = 8.13, *p* = 0.010]. The main effect of *stimulation* was non-significant [*F*_(1, 42)_ = 0.99, *p* = 0.332] and the factor *group* turned out as a trend [*F*_(1, 20)_ = 3.32, *p* = 0.083]. In order to disentangle the significant stimulation^*^group interaction *post hoc t*-tests were applied. Independent samples *t*-tests confirmed a significant difference between the above and below median pitch span group after sham stimulation [*t*_(20)_ = 3.37, *p* = 0.003] whereas the two groups did not differ anymore after cathodal stimulation [*t*_(20)_ = 0.35, *p* = 0.731]. Furthermore, a paired-samples *t*-test showed significantly decreased span performances after cathodal stimulation (*M* = 4.30 ± 0.98) compared to sham stimulation (*M* = 5.50 ± 1.20) in the above median group [*t*_(10)_ = 2.96, *p* = 0.014] (Table 2, Figure 1). In the below median group the comparison between the pitch span performance after sham stimulation (*M* = 3.90 ± 1.01) and cathodal stimulation (*M* = 4.48 ± 1.42) was non-significant [*t*_(10)_ = 1.22, *p* = 0.250] even though this group showed a descriptive improvement following cathodal tDCS (Table 2, Figure 1).

**Table 2:** Memory performance for the tDCS sessions: overview of the overall memory span performances and separated based on a median split of the pitch span performance in the pre-test (below and above median).

	**Overall**	**Below median**	**Above median**
**PITCH SPAN**			
sham tDCS	4.70 ± 1.36	3.90 ± 1.01	**5.50 ± 1.20**
cathodal tDCS	4.39 ± 1.20	4.48 ± 1.42	**4.30 ± 0.98**
**VISUAL SPAN**			
sham tDCS	7.25 ± 1.63	7.24 ± 1.81	7.27 ± 1.53
cathodal tDCS	7.28 ± 1.47	7.06 ± 1.46	7.51 ± 1.52

*A significant deterioration of pitch memory performance after cathodal compared to sham tDCS was revealed for the above median performers, only (bold values)*.

**Figure 2:**
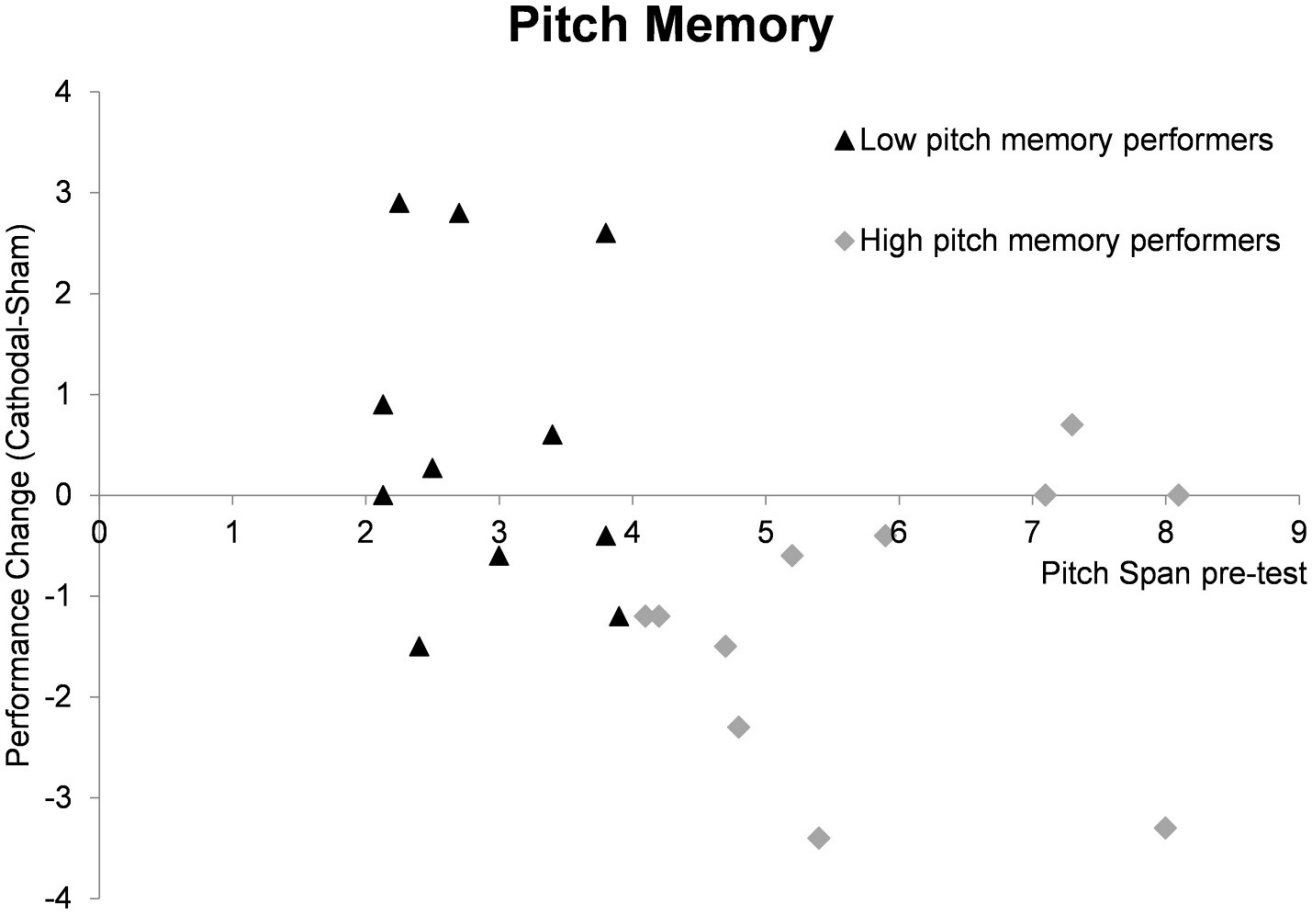
Summary of pitch memory performance change (cathodal tDCS pitch span—sham tDCS pitch span) depending on pre-test memory abilities. The individual data points reflect the pitch memory change after cathodal compared to sham tDCS for individual subjects. In the high pitch memory performers the mean change differed significantly from zero (*p* = 0.014) reflecting a significant inhibitory tDCS effect in this group. The performance change in the low pitch memory performers is more heterogonous and a non-significant results was revealed (*p* = 0.250).

As a next step we calculated the performance change after cathodal tDCS by subtracting the sham memory span scores from the cathodal memory span scores. We then tested whether the performance change differed significantly from zero for both groups in order to reveal a significant modulation effect. In the high pitch memory performance, a significant result was revealed [*t*_(10)_ = 2.96, *p* = 0.014] with mean performance change of −1.20 (±1.34) tones. For the low pitch memory performers the result was non-significant (*p* = 0.250). Figure 2 gives an overview of performance change for every individual subject.

For the visual memory span task a mixed-design ANOVA with *stimulation* (sham vs. cathodal) as the within-subject variable and *group* (above vs. below median pitch span) as the between-subject variable was calculated. Here the main effects as well as the interaction turned out non-significant (*p* > 0.428) indicating that the stimulation had no effect on visual memory performance.

### Pitch perception tasks

The analyses regarding the pitch discrimination tasks (detection and direction task) showed no significant effects of *stimulation* (sham vs. cathodal tDCS). The sample displayed pitch detection thresholds of *M* = 0.24 ± 0.16 (sham tDCS) and *M* = 0.19 ± 0.10 (cathodal tDCS) and an paired samples *t*-test revealed a non-significant result [*t*_(21)_ = 1.24, *p* = 0.227]. The thresholds for pitch direction were *M* = 0.31 ± 0.41 (sham) and *M* = 0.46 ± 0.60 (cathodal) and the difference was also non-significant, *t*_(21)_ = 1.01, *p* = 0.326. Furthermore, mixed-design ANOVAs with *stimulation* (sham vs. cathodal tDCS) and *group* (above vs. below median pitch span) on the pitch detection and direction task respectively showed non-significant effects. Independent-samples *t*-tests between the above and below median pitch memory group revealed that there were no differences on either task or stimulation condition (*p*-values > 0.175).

## Discussion

The aim of the present study was to explore the causal significance of the right DLPFC for pitch memory in non-musicians. Pitch and visual memory spans were compared after participants received either sham or cathodal tDCS. For the overall sample no effect of stimulation on pitch memory performances was revealed. When taking pre-test performance into account it was revealed that cathodal tDCS led to a significant decline in pitch memory in good pitch memory performers only. Low pitch memory performers showed a descriptive but non-significant facilitation of pitch memory after cathodal tDCS. Modulation effects were neither shown for the visual memory span task nor for the pitch perception tasks. The results highlight that the right DLPFC is causally involved in the pitch memory process and interestingly, the study revealed a differential and selective effect of tDCS depending on pre-test pitch memory abilities.

The data provide evidence for a causal significance of the right DLPFC for pitch memory in non-musicians, even though a significant modulation effect was only revealed in good pitch memory possessors. The present findings support previous neuroimaging studies highlighting the activation of the right inferior frontal lobe during pitch memory processes (Zatorre et al., 1994; Gaab et al., 2003; Kumar et al., 2016). They are also in line with a previous tACS study of our group where we could show that 35 Hz tACS over the right DLPFC of amusic participants led to a significant improvement in their pitch memory performance. Thus, highlighting a functional relationship between the right DLPFC and pitch memory abilities (Schaal et al., 2015c). The present study used a reverse engineering approach and showed that disrupting the pitch memory process in non-musicians by applying cathodal tDCS over the right DLPFC led to significantly impaired pitch memory spans in good pitch memory performers. No modulation effects were observed for the visual control task, supporting the specific involvement of the right DLPFC for auditory pitch memory functions (Schaal et al., 2015c). As the prefrontal lobe is closely connected to auditory brain areas, the significance of prefrontal areas for auditory functions has been highlighted (Plakke and Romanski, 2014; Elmer et al., 2015; Kumar et al., 2016) and—in line with the present data—the DLPFC has been shown to be involved in auditory memory functions (Bodner et al., 1996; Fiez et al., 1996; Opitz et al., 2000; Strand et al., 2008). The exact role of the DLPFC for the musical memory process has not been investigated yet. At this point one can only speculate whether the significance of the right DLPFC for pitch memory is linked to a specific memory stage. On the basis of sparse neuroimaging studies, it may be hypothesized that the DLPFC is predominantly involved in the encoding process of the memory information (Opitz et al., 2000; Bor et al., 2004). In order to make a more reliable statement, it would be desirable to use transcranial magnetic stimulation in order to disrupt the pitch memory process at the different memory stages (encoding, retention, retrival) of the pitch memory task used in the present study. Such an approach would allow to explore if the DLPFC is significantly involved in one or more stages of the memory process. Along these lines a previous study of our group could show that stimulation over the left supramarginal gyrus during the retention phase of a pitch memory paradigm led to decreased performance while stimulation during retrieval did not show a modulatory effect (Schaal et al., 2015d).

The present study highlights the involvement of the right DLPFC for pitch memory. However, this is only one area of a complex neural network of frontal, parietal and temporal areas, which has been shown to be involved in pitch memory processes (Zatorre et al., 1994; Gaab et al., 2003). It might also be possible that the applied stimulation over the right DLPFC also affected connecting brain areas. However, it is important to keep in mind that we can only speculate on the possible remote effects of tDCS as we did not include any brain imaging techniques. It might be that cathodal tDCS over the right DLPFC did not only suppress the activity in the target area but also disrupted the connectivity between frontal and temporal areas. Several studies have investigated the activation and morphology of the arcuate fasciculus, a fiber tract which connects frontal and temporal areas, for auditory memory and have shown that abnormalities in this brain region are linked to congenital amusia and consequently to impaired pitch memory abilities (Loui et al., 2009; Hyde et al., 2011). Thus, it can be hypothesized that remote effects of the stimulation input also affected this region.

The results of this study highlight that pre-test performances influence the modulatory effect of tDCS. Similarly, previous studies have revealed differential effects of tDCS for groups with low and high baseline performances on several tasks (Benwell et al., 2015; Hsu et al., 2016; McConathey et al., 2017; Pollok et al., 2017). For example, McConathey et al. (2017) investigated the effect of sham and anodal tDCS over the left prefrontal cortex on language skills in participants with primary progressive aphasia. Their data showed that participants who performed low in the language tasks at baseline showed improvements in more subtests and to a greater extent than participants who performed better at baseline. The authors claimed that the level of baseline performance could predict whether participants would respond positively to the stimulation or not. Taken together, the results by McConathey et al. (2017) and our results would suggest that the participants with a higher potential to display the expected modulatory change are effected by the stimulation applied, i.e. in our case it is more likely that good performers show a deterioration in performance and in the study by McConathey et al. (2017) the low performing group have a greater range to improve. A systematic investigation of this assumption is desirable to better understand the influence of baseline performances on stimulation effects.

Looking at the pre-test pitch performances of our sample, it is notable that a group of participants (below median group) displayed fairly poor pitch memory abilities while the above median group displayed pitch memory abilities which match the performance level of healthy non-musicians reported in previous studies (Williamson and Stewart, 2010; Schaal et al., 2013, 2015b,c, 2017). In the above median performers, cathodal tDCS over the right DLPFC selectively led to a significant deterioration of pitch memory supporting neuroimaging studies which have highlighted the activation of the right inferior frontal lobe during pitch memory processes (Zatorre et al., 1994; Gaab et al., 2003; Albouy et al., 2013). This result is in line with our hypothesis that cathodal tDCS leads to a suppression of neural activity in the right DLPFC and consequently results in a deterioration of pitch memory performance. The directional effect fits well to previous research, which has shown that cathodal tDCS over the left SMG leads to diminished pitch memory abilities in non-musicians (Vines et al., 2006; Schaal et al., 2015b).

For the non-musicians displaying low pitch memory abilities in the pre-test (below median group), we saw that cathodal tDCS did not impair pitch memory performance but descriptively facilitated the pitch memory span even though this effect was non-significant. It should be highlighted that performance levels of this group were unexpectedly low. Although the question why some participants performed below the expectations remains fairly open, it is unlikely that a lack of motivation caused the low pitch memory performance as low memory performances were restricted to the auditory memory domain. On the visual span task, low and high pitch memory groups performed equally well. On the other hand, one might raise a plea that the below median group could be considered as amusic as the pitch memory span was extremely low whereas the visual memory span was not. Even though this cannot be ruled out completely, we would argue that it is fairly unlikely since the results of the pitch perceptual tasks were comparable between the below and above median pitch memory group. If the below median group would consist of amusic individuals impairments should be present, especially in the pitch direction task (Williamson and Stewart, 2010; Schaal et al., 2015c). Nevertheless, it would be desirable for future studies to include the Montreal Battery of Evaluation of Amusia (Peretz et al., 2003) in order to completely rule out that amusic participants take part and to ensure a more homogeneous group. Investigating whether tDCS over the right DLPFC in congenital amusics would influence pitch memory performance and may even result in a significant improvement of performances is of further interest.

In sum, the present study revealed a defined involvement of the right DLPFC for pitch memory in non-musicians, as the results show that cathodal tDCS over the right DLPFC led to significantly impaired pitch memory performance in good pitch memory possessors. Visual memory performance was not affected by tDCS. The study highlights the importance of baseline abilities for detecting modulatory effects of tDCS.

## Author contributions

NS: conception and design of the experiment, data collection and analyses, interpretation of the data, drafting the article; MK: data collection, interpretation of the data, critical revision of the article; AK and VK: interpretation of the data, critical revision of the article; JP: conception and design of the experiment, critical revision of the article; BP: conception and design of the experiment, analysis and interpretation of the data, critical revision of the article.

### Conflict of interest statement

The authors declare that the research was conducted in the absence of any commercial or financial relationships that could be construed as a potential conflict of interest.
